# Commensal Microbiota Effects on Craniofacial Skeletal Growth and Morphology

**DOI:** 10.1002/jbm4.10775

**Published:** 2023-05-31

**Authors:** Joy E. Gerasco, Jessica D. Hathaway‐Schrader, Nicole A. Poulides, Matthew D. Carson, Naoto Okhura, Caroline Westwater, Nan E. Hatch, Chad M. Novince

**Affiliations:** ^1^ Department of Oral Health Sciences, College of Dental Medicine Medical University of South Carolina Charleston SC USA; ^2^ Department of Stomatology‐Division of Periodontics, College of Dental Medicine Medical University of South Carolina Charleston SC USA; ^3^ Department of Pediatrics‐Division of Endocrinology, College of Medicine Medical University of South Carolina Charleston SC USA; ^4^ Department of Orthodontics, Adam's School of Dentistry University of North Carolina Chapel Hill NC USA; ^5^ Department of Orthodontics and Pediatric Dentistry, School of Dentistry University of Michigan Ann Arbor MI USA; ^6^ Department of Microbiology and Immunology, College of Medicine Medical University of South Carolina Charleston SC USA

**Keywords:** BONE–MICROBIOTA INTERACTORS, CRANIOMETRY, PRECLINICAL STUDIES

## Abstract

Microbes colonize anatomical sites in health to form commensal microbial communities (e.g., commensal gut microbiota, commensal skin microbiota, commensal oral microbiota). Commensal microbiota has indirect effects on host growth and maturation through interactions with the host immune system. The commensal microbiota was recently introduced as a novel regulator of skeletal growth and morphology at noncraniofacial sites. Further, we and others have shown that commensal gut microbes, such as segmented filamentous bacteria (SFB), contribute to noncraniofacial skeletal growth and maturation. However, commensal microbiota effects on craniofacial skeletal growth and morphology are unclear. To determine the commensal microbiota's role in craniofacial skeletal growth and morphology, we performed craniometric and bone mineral density analyses on skulls from 9‐week‐old female C57BL/6T germ‐free (GF) mice (no microbes), excluded‐flora (EF) specific‐pathogen‐free mice (commensal microbiota), and murine‐pathogen‐free (MPF) specific‐pathogen‐free mice (commensal microbiota with SFB). Investigations comparing EF and GF mice revealed that commensal microbiota impacted the size and shape of the craniofacial skeleton. EF versus GF mice exhibited an elongated gross skull length. Cranial bone length analyses normalized to skull length showed that EF versus GF mice had enhanced frontal bone length and reduced cranial base length. The shortened cranial base in EF mice was attributed to decreased presphenoid, basisphenoid, and basioccipital bone lengths. Investigations comparing MPF mice and EF mice demonstrated that commensal gut microbes played a role in craniofacial skeletal morphology. Cranial bone length analyses normalized to skull length showed that MPF versus EF mice had reduced frontal bone length and increased cranial base length. The elongated cranial base in MPF mice was due to enhanced presphenoid bone length. This work, which introduces the commensal microbiota as a contributor to craniofacial skeletal growth, underscores that noninvasive interventions in the gut microbiome could potentially be employed to modify craniofacial skeletal morphology. © 2023 The Authors. *JBMR Plus* published by Wiley Periodicals LLC on behalf of American Society for Bone and Mineral Research.

## Graphical abstract

Comparison of germ‐free mice (no microbes) to excluded‐flora mice (commensal microbiota) showed that commensal microbiota influenced craniofacial skeletal growth and morphology (*blue arrows denote commensal microbiota‐induced changes). Comparison of excluded‐flora mice (commensal microbiota) to murine‐pathogen‐free mice (commensal microbiota with segmented filamentous bacteria [SFB]) revealed that specific commensal gut microbes contributed to craniofacial skeletal growth and morphology (*orange arrows denote SFB‐induced changes).

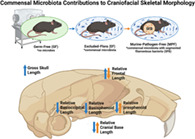

## Introduction

Nonpathogenic microbes colonize host anatomic barrier sites to form commensal microbiota communities (e.g., commensal gut microbiota, commensal skin microbiota, commensal oral microbiota).^[^ ^1^, ^2^, ^3^, ^4^ ^]^ Commensal microbiota interactions with the host direct the development of immunity, which has indirect effects on host growth and maturation.^[^ ^5^, ^6^, ^7^, ^8^ ^]^ The commensal microbiota was recently introduced as a critical regulator of skeletal growth and morphology at noncraniofacial sites.^[^ ^9^, ^10^, ^11^, ^12^, ^13^, ^14^ ^]^ Moreover, our group and others have shown that commensal gut microbes, such as segmented filamentous bacteria (SFB), contribute to noncraniofacial skeletal growth and maturation.^[^ ^13^, ^14^ ^]^

The craniofacial skeleton is a unique skeletal complex that derives from the neural crest and mesoderm and develops through both intramembranous and endochondral ossification.^[^ ^15^, ^16^ ^]^ The craniofacial skeleton houses the brain and sensory organs for vision, hearing, taste, and smell, supports mastication and speaking, and impacts appearance and psychosocial well‐being. Therefore, mechanisms regulating craniofacial growth and morphology are important.^[^ ^17^, ^18^ ^]^ Murine models have been extensively used to study normal craniofacial development^[^ ^19^, ^20^, ^21^ ^]^ and craniofacial malformations caused by genetics, teratogens, and environmental factors.^[^ ^22^, ^23^, ^24^ ^]^ However, the role of commensal microbiota in physiologic craniofacial skeletal growth and morphology is unknown.

To define the role of commensal microbiota in normal craniofacial skeletal growth and morphology, we performed studies comparing germ‐free (GF) mice, excluded‐flora (EF) mice, and murine‐pathogen‐free (MPF) mice. GF mice, which are devoid of all microbes, are reared in sterile isolators. Specific‐pathogen‐free mice, which are colonized by commensal microbiota, are reared in ventilated isolators within barrier facilities that validate specific microbes are not present. Taconic Biosciences animal health barrier facilities provided the opportunity to acquire specific‐pathogen‐free mice that differed by the known colonization status of SFB. EF mice are specific‐pathogen‐free mice that are devoid of SFB, and MPF mice are specific‐pathogen‐free mice that harbor SFB. SFB are commensal gut bacteria that colonize the ileum following weaning.^[^ ^25^, ^26^, ^27^ ^]^ SFB colonization critically influences commensal microbiota actions on host immunity and development.^[^ ^13^, ^14^, ^27^, ^28^, ^29^, ^30^ ^]^ Therefore, comparing specific‐pathogen‐free mice that differ by SFB colonization status provides insights about the role of commensal gut microbes in commensal microbiota effects on normal growth and morphology.

Cephalometric and craniometric measurements are used in the surgical and dentofacial orthopedic treatment of craniofacial malformations and achieve a more harmonious dentofacial complex.^[^ ^31^, ^32^ ^]^ Craniometry is utilized in preclinical murine model research to advance knowledge about abnormal and healthy craniofacial skeletal growth and development.^[^ ^19^, ^20^, ^21^, ^22^, ^23^, ^24^ ^]^ Herein, we performed craniometric and bone mineral density (BMD) analyses in skulls from 9‐week‐old female C57BL/6T GF mice (no microbes), EF mice (commensal microbiota), and MPF mice (commensal microbiota with SFB). Comparing GF mice to EF mice help define the role of commensal microbiota in normal craniofacial skeletal growth and morphology. Comparing EF mice to MPF mice made it possible to discern commensal gut microbe contributions to physiologic craniofacial skeletal growth and morphology.

## Materials and Methods

### Mice

C57BL/6T GF mice were obtained from Taconic Biosciences (Rensselaer, NY, USA) and were bred and maintained in sterile isolators at the Medical University of South Carolina (MUSC; Charleston, SC, USA) Gnotobiotic Animal Core. GF mice were fed autoclaved LabDiet 5010 (LabDiet, St. Louis, MO, USA). Nine‐week‐old EF mice and MPF mice were obtained from Taconic Biosciences (Rensselaer, NY, USA), where they were bred and maintained in their respective barrier facilities. EF and MPF mice were fed autoclaved NIH‐31M diet (Envigo, Indianapolis, IN, USA). Upon arrival at MUSC, the EF and MPF mice were housed in a specific‐pathogen‐free vivarium and sacrificed within 48 h. All animals were euthanized at 9.0–9.5 weeks of age. Animals were group‐housed four to five per cage. Room temperature and humidity were maintained within the ranges recommended by the *Guide for the Care and Use of Laboratory Animals*.^[^ ^33^ ^]^ Room light:dark cycle was maintained on a 12‐h on:off schedule. Animal experimentation was approved by the MUSC Institutional Animal Care and Use Committee and carried out in accordance with approved guidelines.

### Quantitative real‐time PCR (qRT‐PCR) 16S rDNA analyses

Following euthanasia, ileum contents were collected for bacterial 16S rDNA analysis. Genomic DNA was isolated using Qiagen DNeasy Powersoil Pro Kit (Qiagen, Hilden, Germany), per the manufacturer's instructions. Total DNA was quantified using the Nanodrop 1000 (Thermo Fisher Scientific, Waltham, MA, USA). 16S rDNA was amplified on the StepOnePlus System (Applied Biosystems, Foster City, CA, USA) via a qRT‐PCR reaction protocol using 2× Fast SYBR Green Master Mix (Applied Biosystems) forward/reverse primers (Integrated DNA Technology, Coralville, IA, USA) and DNA template, as described previously.^[^ ^34^, ^35^, ^36^ ^]^ A 30‐cycle qRT‐PCR protocol was utilized; cycle number 25 was the cutoff for nonspecific amplification of the Universal 16S gene.^[^ ^13^, ^37^ ^]^ Bacterial load analysis was conducted in which bacterial load was evaluated by normalizing the Universal 16S gene to a bacterial DNA standard (ZymoBIOMICS; Zymo Research, Irvine, CA, USA), as described previously.^[^ ^13^, ^36^, ^38^ ^]^ Relative quantification of Universal 16S rDNA was carried out by the 2^−ΔCT^ method.^[^ ^39^ ^]^ In the bacterial phyla analysis, phylum‐level outcomes are reported relative to the Universal 16S gene, as described previously.^[^ ^36^, ^38^ ^]^ Relative quantification of phylum‐level rDNA was performed by the 2^−ΔΔCT^ method.^[^ ^40^ ^]^ In SFB analysis, SFB presence was evaluated by normalizing the SFB rDNA gene to a bacterial DNA standard (ZymoBIOMICS; Zymo Research), as described previously.^[^ ^13^, ^41^ ^]^ Relative quantification of SFB rDNA was performed by the 2^−ΔCT^ method.^[^ ^39^ ^]^ Integrated DNA Technologies forward (F)/reverse (R) primer sequences included the following:

Universal 16S^[^ ^34^ ^]^: F = 5′‐AAACTCAAAKGAATTGACGG‐3′; R = 5′‐CTCACRRCACGAGCTGAC‐3′,
Pseudomonadota^[^ ^34^ ^]^: F = 5′‐TCGTCAGCTCGTGTYGTGA‐3′; R = 5′‐CGTAAGGGCCATGATG‐3′,
Actinomycetota^[^ ^34^ ^]^: F = 5′‐TACGGCCGCAAGGCTA‐3′; R = 5′‐TCRTCCCCACCTTCCTCCG‐3′,
Bacteroidota^[^ ^34^ ^]^: F = 5′‐CRAACAGGATTAGATACCCT‐3′; R = 5′‐GGTAAGGTTCCTCGCGTAT‐3′,
Bacillota^[^ ^34^ ^]^: F = 5′‐TGAAACTYAAAGGAATTGACG‐3′; R = 5′‐ACCATGCACCACCTGTC‐3′,
SFB^[^ ^13^, ^41^ ^]^: F = 5′‐GACGCTGAGGCATGAGAGCAT‐3′; R = 5′‐GACGGCACGGATTGTTATTCA‐3′.

### qRT‐PCR mRNA analyses

Ileum specimens were flash‐frozen, pulverized, and homogenized in TRIzol Reagent (Invitrogen, Carlsbad, CA, USA). RNA extraction was performed via the TRIzol method, as reported previously.^[^ ^13^, ^41^ ^]^ Total RNA was quantified via the NanoDrop 1000 (Thermo Fisher Scientific). cDNA was synthesized from RNA using Taqman Random Hexamers and Reverse Transcription Reagents (Applied Biosystems), following the manufacturer's protocol.^[^ ^13^, ^41^ ^]^ mRNA was amplified on the StepOnePlus System (Applied Biosystems) via a qRT‐PCR reaction protocol using TaqMan Fast Advanced Master Mix and primer probes (Applied Biosystems), as described previously.^[^ ^13^, ^41^ ^]^ Relative quantification of mRNA was performed by the 2^−ΔΔCT^ method^[^ ^40^ ^]^; *Gapdh* was used as an internal control gene. The TaqMan primer‐probe assays used included *Gapdh* = Mm99999915_g1; *Il17a* = Mm00439618_m1.

### Tibia microradiography imaging/length analysis

Following euthanasia, tibiae were isolated and fixed in 10% phosphate‐buffered formalin for 24 h at room temperature; specimens were stored in 70% ethanol at 4°C. Ex vivo microradiographs of tibiae were acquired with a Faxitron LX‐60 (Faxtiron X‐ray, Lincolnshire, IL, USA), using the following acquisition parameters: beam energy = 36 kVp, exposure time = 40 s. Tibia length measurements were performed via calibrated microradiograph images, measuring from the intercondylar eminence to the lateral malleolus.

### Skull micro–CT imaging

Following euthanasia, skulls were isolated and fixed in 10% phosphate‐buffered formalin for 24 h at room temperature; specimens were stored in 70% ethanol at 4°C. Mandibles were dissected and skull specimens were scanned with a Scanco Medical micro–CT (μCT) 40 scanner (Scanco Medical; Brüttisellen, Switzerland), using the following acquisition parameters: X‐ray tube potential = 70 kVp, X‐ray intensity = 55 μA, integration time = 200 ms, and isotropic voxel size = 18 μm^3^. Calibrated three‐dimensional (3D) images of skulls were reconstructed for craniometric measurements and BMD analyses.

### μCT craniometric analyses

Skull craniometric measurements were performed using Analyze 14.0 Bone Microarchitecture Analysis software (Analyze Direct, Seattle, WA, USA). Linear measurements were carried out on reoriented 3D μCT reconstructions, with a fixed threshold of 2500 Hounsfield units (HU). For frontal and parietal bones, linear measurements were taken bilaterally and averaged. Linear measurements were performed three separate times for each outcome of interest, and the arithmetic mean of these measurements is reported for each biological replicate. Skull length (nasale to paro) is reported as a direct measurement. All other linear measurements were normalized to skull length (nasale to paro) to account for skull size differences, as reported previously.^[^ ^42^, ^43^, ^44^ ^]^ Landmarks for craniometric analyses are depicted via schematics: skull length, skull width, and skull height (Fig. S1); skull length, nasal bone length, cranial vault bone lengths, and cranial base bone lengths (Fig. S1). Studies were performed and are reported in accordance with guidelines for assessment of bone microstructure in rodents using μCT.^[^ ^45^ ^]^

### μCT bone mineral density analyses

Skull BMD measurements were acquired using Analyze 14.0 Bone Microarchitecture Analysis software (Analyze Direct). BMD analyses were carried out using reoriented 3D μCT reconstructions. A fixed threshold of 2000 HU was used to discern mineralized tissue. BMD analysis was performed in the following volumes of interest, adapted from methods described previously^[^ ^21^, ^46^, ^47^ ^]^: *Basioccipital bone analysis*: 900 μm^3^ volume of interest (VOI) positioned 180 μm posterior to the junction of the basisphenoid and basioccipital bones, the spheno‐occipital synchondrosis (SOS). The VOI was centered mediolaterally across the median sagittal plane. *Basisphenoid bone analysis*: 900 μm^3^ VOI positioned 360 μm anterior to the SOS and 360 μm superior from the inferior extent of the pterygoid bone/process at the intersection of the SOS. *Presphenoid bone analysis*: 900 μm^3^ VOI positioned 270 μm anterior to the junction of the basisphenoid and presphenoid bones, the intersphenoid synchondrosis (ISS). The VOI was oriented in the superior–inferior and mediolateral directions centered on the presphenoid bone. *Frontal bone analysis*: 1440 μm^3^ VOI was analyzed at the left frontal bone. The VOI was positioned 720 μm lateral to the sagittal suture and 1440 μm anterior to the coronal suture. *Parietal bone analysis*: 1440 μm^3^ VOI was analyzed at the left parietal bone. The VOI was positioned 1440 μm lateral to the sagittal suture and centered between the lambdoid and coronal sutures in the anterior–posterior plane. *Interparietal bone analysis*: 900 μm^3^ VOI positioned 900 μm anterior to the junction of the interparietal and occipital bones. The VOI was centered mediolaterally across the median sagittal plane. Landmarks and VOIs for BMD analysis are depicted via schematics: cranial vault bones (Fig. S2*A*) and cranial base bones (Fig. S2*E*). Studies were performed and are reported in accordance with guidelines for assessment of bone microstructure in rodents using μCT.^[^ ^45^ ^]^

### Statistical analysis

Statistical analysis was carried out using GraphPad Prism version 9.0 (GraphPad, La Jolla, CA, USA). Unpaired two‐tailed *t*‐test (*p* < 0.05) was performed comparing 16S rDNA analysis outcomes in EF versus MPF mice. One‐way ANOVA (*α* = 0.05) with Tukey post hoc test (*p* < 0.05) was carried out comparing outcomes in GF, EF, and MPF mice for all other studies. When unpaired *t* test was used, *F* test was performed to validate the data come from populations that are Gaussian and have equal variances. When one‐way ANOVA was utilized, a Brown–Forsythe test and Levene test were carried out to validate the data come from populations that are Gaussian and have equal variances. The Shapiro–Wilk test (*α* = 0.05) was applied to all data sets to determine whether the data came from normally distributed populations. In the case that the Shapiro–Wilk test did not validate that a data set was normally distributed, the ROUT outlier test (*Q* = 0.1%) was performed to remove definitive outliers. Data are plotted as box and whisker plots that display all data points, the interquartile range (height of the box), median (internal horizontal bar), arithmetic mean (“plus” sign), and maximum and minimum values (external upper and lower bars). Power analysis was performed based on the authors' prior experience investigating skeletal morphology in mice and was carried out in consultation with the MUSC Bioinformatics Core Biostatistical Unit.

## Results

### Commensal microbiota does not alter somatic growth in 9‐week‐old female C57BL/6T mice

**Fig. 1:**
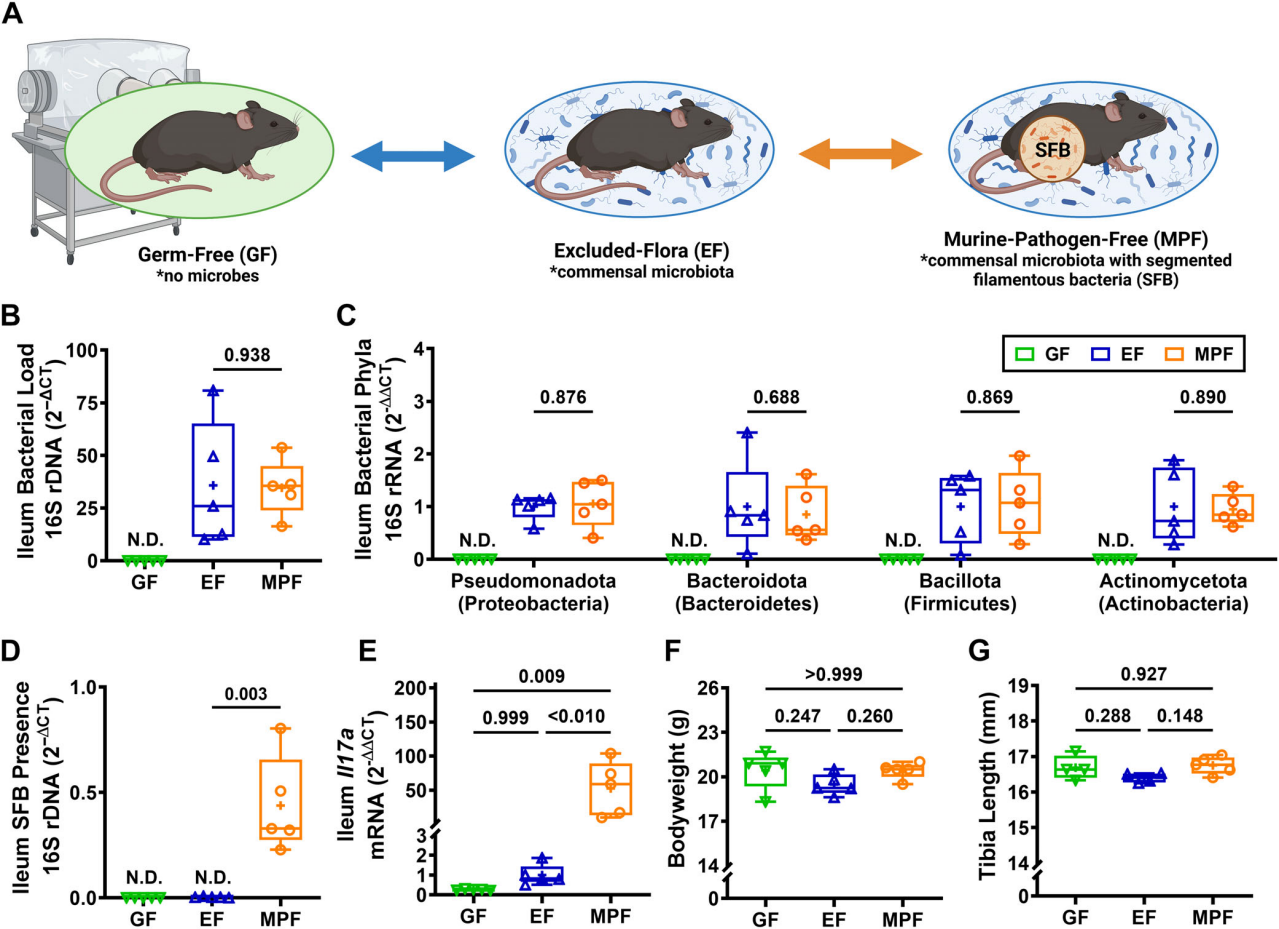
Commensal microbiota do not alter somatic growth in 9‐week‐old female C57BL/6T mice. (*A*) Female C57BL/6T GF mice (no microbes), EF mice (commensal microbiota), and MPF mice (commensal microbiota with SFB) were euthanized at age 9 weeks; specimens were isolated for analyses. (*A*) Schematic of study design. (*B*–*D*) qRT‐PCR 16S rDNA analysis of ileum contents (*n* = 5/group) evaluating (*B*) bacterial load, (*C*) phyla, and (*D*) SFB presence. (*B*) Bacterial load determined by normalizing the Universal 16S gene to a bacterial DNA standard; quantification via the 2^−ΔCT^ method. (*C*) Phylum outcomes determined by normalizing phylum 16S genes to the Universal 16S gene; quantification by the 2^−ΔΔCT^ method. (*D*) SFB presence determined by normalizing the SFB 16S gene to a bacterial DNA standard; quantification via the 2^−ΔCT^ method. (*E*) qRT‐PCR mRNA analysis of *Il17a* in ileum (*n* = 5/group); relative quantification of mRNA was performed via the 2^−ΔΔCT^ method. (*F*) Body weight analysis (*n* = 5/group). (*G*) Tibia length analysis (*n* = 4–5/group). (*B*–*D*) Unpaired two‐tailed *t*‐test (*p* value <0.05) was performed comparing 16S rDNA analysis outcomes in EF versus MPF mice. (*E*, *F*) One‐way ANOVA (*α* = 0.05) with Tukey post hoc test (*p* value <0.05) was carried out comparing outcomes in GF, EF, and MPF mice.

We compared 9‐week‐old female C57BL/6T GF mice (no microbes) and EF mice (commensal microbiota) to determine the role of commensal microbiota in craniofacial skeletal growth and morphology (Fig. 1*A*). We compared 9‐week‐old female C57BL/6T EF mice (commensal microbiota) and MPF mice (commensal microbiota with SFB) to delineate whether commensal gut microbes contributed to commensal microbiota effects on craniofacial skeletal growth and morphology (Fig. 1*A*).

16S rDNA analyses were performed with ileum contents to validate the murine gut microbiome phenotype (Fig. 1*B–D*). Bacteria were not detectable in GF mice (Fig. 1*B–D*). The bacterial load was similar (Fig. 1*B*) and bacterial phyla expression was not different (Fig. 1*C*) in EF versus MPF mice. SFB was detected in MPF mice, but not EF mice (Fig. 1*D*). Consistent with the presence of SFB, which induces T_H_17/IL17A immunity,^[^ ^27^, ^28^ ^]^ *Il17a* mRNA was greater than 50 times higher in the ileum of MPF versus EF mice (Fig. 1*E*).

We evaluated alterations in body weight and tibia length to determine commensal microbiota effects on somatic (body) growth outcomes. Body weight measurements were performed immediately prior to euthanasia. Tibia length measurements were carried out on ex vivo microradiographs of tibiae. Body weight was similar (Fig. 1*F*) and tibia length was not different (Fig. 1*G*) in GF versus EF mice or EF versus MPF mice. These data support the idea that commensal microbiota does not alter somatic growth outcomes in 9‐week‐old female C57BL6/T mice.

### Commensal microbiota impacts craniofacial skeletal morphology in 9‐week‐old female C57BL/6T mice

**Fig. 2:**
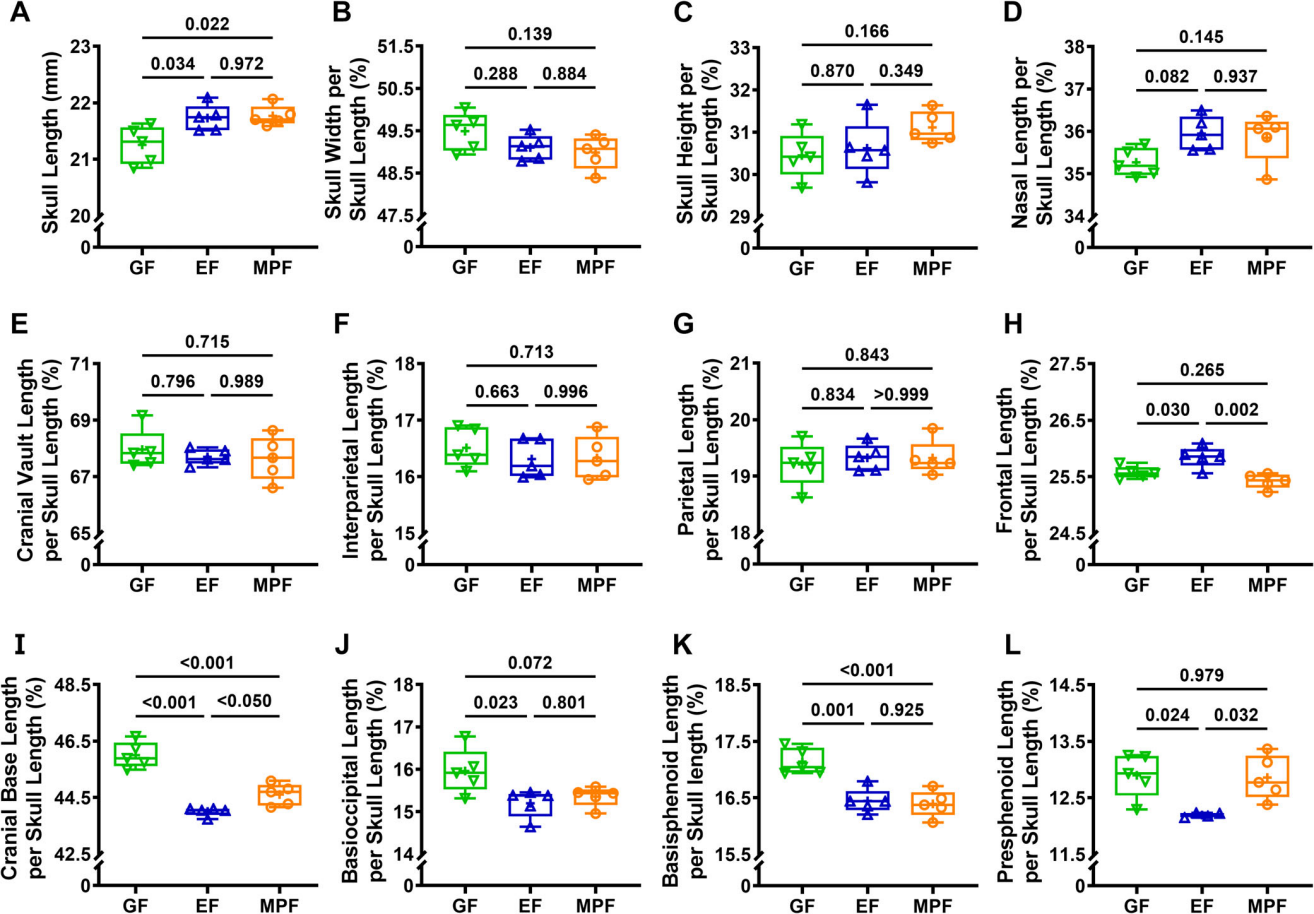
Commensal microbiota impact craniofacial skeletal morphology in 9‐week‐old female C57BL/6T mice. Female C57BL/6T GF mice (no microbes), EF mice (commensal microbiota), and MPF mice (commensal microbiota with SFB) were euthanized at age 9 weeks; skulls were isolated for analyses (*n* = 5/group). Craniometric measurements were performed using μCT 3D reconstructions of the skull and standard craniometric landmarks (Fig. S1): skull length (*A*); skull width per skull length (*B*); skull height per skull length (*C*); nasal bone length per skull length (*D*); cranial vault length per skull length (*E*); interparietal bone length per skull length (*F*); parietal bone length per skull length (*G*); frontal bone length per skull length (*H*); cranial base length per skull length (*I*); basioccipital bone length per skull length (*J*); basisphenoid bone length per skull length (*K*); presphenoid bone length per skull length (*L*). One‐way ANOVA (*α* = 0.05) with Tukey post hoc test (*p* < 0.05) was carried out comparing outcomes in GF, EF, and MPF mice.

We performed craniometric measurements on reconstructed 3D μCT images of skulls to evaluate commensal microbiota effects on craniofacial skeletal growth and morphology (Figs. 2 and S1). Skull length (Fig. 2*A*) is reported as a direct measurement. Skull width (Fig. 2*B*), skull height (Fig. 2*C*), nasal bone length (Fig. 2*D*), cranial vault bone lengths (Fig. 2*E–H*), and cranial base bone lengths (Fig. 2*I–L*) are reported relative to skull length.

The direct skull length was increased in EF versus GF mice (Fig. 2*A*), which demonstrates that commensal microbes influence the skull length. The direct skull length was similar in MPF versus EF mice (Fig. 2*A*), which implies that commensal gut microbes do not contribute to skull length. Skull width per skull length (Fig. 2*B*), skull height per skull length (Fig. 2*C*), and nasal bone length per skull length (Fig. 2*D*) were not different in EF versus GF mice or MPF versus EF mice. These data support the idea that the commensal microbiota does not influence the relative skull width, skull height, or nasal bone length in 9‐week‐old female C57BL/6T mice.

Cranial vault bone length analyses (Fig. 2*E–H*) demonstrated that the cranial vault length per skull length was not different in EF versus GF mice or MPF versus EF mice (Fig. 2*E*). The similar relative cranial vault lengths found in EF versus GF mice and MPF versus EF mice (Fig. 2*E*) were attributed to a lack of alterations in interparietal (Fig. 2*F*) and parietal (Fig. 2*G*) bone lengths per skull length. Interestingly, the frontal bone length per skull length was increased in EF versus GF mice and reduced in MPF versus EF mice (Fig. 2*H*). The increased relative frontal bone length detected in EF versus GF mice (Fig. 2*H*) suggests that the commensal microbiota affects cranial vault growth and morphology. The reduced relative frontal bone length detected in MPF versus EF mice (Fig. 2*H*) supports the idea that commensal gut microbes play a role in commensal microbiota effects on cranial vault growth and morphology.

Cranial base bone length analyses (Fig. 2*I–L*) showed the cranial base length per skull length was decreased in EF versus GF mice (Fig. 2*I*). The shortened relative cranial base length in EF versus GF mice (Fig. 2*I*) was attributed to EF mice having reduced basioccipital (Fig. 2*J*), basisphenoid (Fig. 2*K*), and presphenoid (Fig. 2*L*) bone lengths per skull length. Cranial base craniometric findings in EF versus GF mice suggest that the commensal microbiota influences cranial base growth and morphology. Cranial base analyses comparing MPF versus EF mice showed that MPF mice exhibited an increased cranial base length per skull length (Fig. 2*I*). The elongated relative cranial base length in MPF versus EF mice (Fig. 2*I*) was due to MPF mice having an enhanced presphenoid bone length per skull length (Fig. 2*L*). Cranial base craniometric study outcomes in MPF versus EF mice support the idea that commensal gut microbes contribute to commensal microbiota effects on cranial base growth and morphology.

### Commensal microbiota does not affect cranial bone mineral density in 9‐week‐old female C57BL/6T mice

We carried out BMD analyses in the reconstructed 3D μCT images of skulls to elucidate commensal microbiota effects on bone mass in the cranial vault and cranial base. Cranial vault analyses included the interparietal, parietal, and frontal bones (Fig. S2*A–D*), and cranial base analyses included the basioccipital, basisphenoid, and presphenoid bones (Fig. S2*E–H*). Cranial vault BMD outcomes were not different in EF versus GF mice or MPF versus EF mice (Fig. S2*A–D*). Furthermore, cranial base BMD outcomes were similar in EF versus GF mice and MPF versus EF mice (Fig. S2*E–H*). BMD study findings support the idea that the commensal microbiota does not critically impact bone mass accrual in the cranial vault or cranial base of 9‐week‐old female C57BL/6T mice.

## Discussion

This study comparing 9‐week‐old female C57BL/6T mice reared under defined barrier conditions revealed that the commensal microbiota plays a role in craniofacial skeletal growth and morphology. Results comparing GF mice to EF mice showed that the commensal microbiota can enlarge the gross skull length and restrict the relative cranial base length. Results comparing EF mice to MPF mice, which differ by SFB colonization status, demonstrated that MPF mice exhibited an elongated relative cranial base length. These findings support the idea that commensal gut microbes contribute to craniofacial skeletal growth and morphology.

Comparing GF mice to EF mice demonstrated that the commensal microbiota attenuated the relative cranial base length owing to decreased relative basioccipital, basisphenoid, and presphenoid bone lengths. Appreciating that cranial base bones form through endochondral ossification, these findings are in line with prior studies showing that the commensal microbiota regulates endochondral bone formation processes at nonoral skeletal sites.^[^ ^9^, ^10^, ^11^, ^12^, ^13^, ^14^ ^]^ Interestingly, EF versus GF mice exhibited an increased relative length in the frontal bone. The frontal bone forms through intramembranous ossification, suggesting that commensal microbiota also influences intramembranous bone formation processes.

While this initial report was centered on craniometry, future studies should evaluate suture and synchondrosis involvement. This is important because sutures and synchondroses contribute to craniofacial skeletal growth and morphology. Moreover, studies are needed to further assess commensal microbiota effects on the cranial base. The cranial base plays an extremely important role in total skull morphology as the growth site for the skull is located at the ISS.^[^ ^48^ ^]^ The cranial base develops earlier than the face or cranial vault and has long been thought to provide patterning and instructions for the final form of the overall skull.^[^ ^49^ ^]^ Several studies have suggested that cranial base length can predict specific malocclusions in adult life.^[^ ^50^ ^]^ Additionally, alterations in the cranial base angle have been shown to cause skeletal malocclusions.^[^ ^51^ ^]^

We investigated the 9‐week‐old time point because craniofacial skeletal growth peaks around postnatal day 60 in female C57BL/6T mice.^[^ ^21^ ^]^ Future investigations are needed to evaluate earlier time points to define critical postnatal periods where the commensal microbiota contributes to craniofacial skeletal growth and morphology. We utilized μCT to discern commensal microbiota effects on craniometric and BMD outcomes in the postnatal craniofacial skeleton. Ongoing studies are necessary that use histology to delineate cellular and molecular mechanisms mediating commensal microbiota actions on growth and morphology in the craniofacial skeletal complex.

Prior work by our group and others established that commensal microbiota effects on noncraniofacial bone growth and morphology were influenced by mouse strain genetic determinants. Whereas the commensal microbiota impairs bone mass accrual and microarchitecture in long bones of growing C57BL/6 mice,^[^ ^9^, ^10^ ^]^ the commensal microbiota promotes bone formation and longitudinal growth in long bones of growing BALB/c^[^ ^11^ ^]^ and CB6F1 mice.^[^ ^12^ ^]^ Study outcomes reported herein revealed that the commensal microbiota promoted gross skull length and restricted relative cranial base length in C57BL/6 mice. Future research is necessary to determine whether commensal microbiota effects on craniofacial skeletal growth and morphology differ across murine strains.

GF mice were fed LabDiet 5010, and specific‐pathogen‐free mice (EF, MPF mice) were fed NIH‐31M diet. LabDiet 5010 and NIH‐31M are standard hard pellet rodent diets, but they have minor differences in composition and may differ in hardness/consistency. Knowing that administering a soft versus hard diet can affect dentofacial growth outcomes,^[^ ^52^, ^53^ ^]^ a potential study design weakness is that GF and specific‐pathogen‐free mice were not administered the same diet. However, all experimental groups were fed a hard diet, so minor differences in diet consistency are not expected to influence outcomes as dramatically as if the groups were fed soft versus hard diets. Additionally, comparing EF versus MPF mice (both fed NIH‐31M) demonstrated alterations in craniofacial skeletal outcomes, which supports the idea that commensal microbiota effects on craniofacial morphology are independent of diet. However, it will be important for future studies to utilize the same diet across GF and specific‐pathogen‐free animal groups to eliminate diet as a potentially confounding variable.

Investigations in GF animal models have demonstrated that the commensal microbiota supports normal brain development and behavior.^[^ ^54^, ^55^, ^56^, ^57^ ^]^ The growth of the brain, which is housed in the skull, is a major determinant of craniofacial growth and morphology.^[^ ^58^, ^59^, ^60^, ^61^, ^62^ ^]^ Therefore, commensal microbiota actions supporting normal brain development could contribute to the growth and morphology of the craniofacial skeleton through an increase in cranial capacity. This highlights the need for future research elucidating whether commensal microbiota regulation of brain development plays a role in commensal microbiota actions on craniofacial skeletal growth and morphology.

This investigation comparing MPF versus EF mice, which differ by SFB colonization status, supports the idea that commensal gut microbes play a role in the growth and morphology of the craniofacial skeleton. SFB is a commensal gut bacterium that colonizes the ileum after weaning.^[^ ^25^, ^26^, ^27^ ^]^ SFB colonization has broad innate and adaptive immunostimulatory effects, including the induction of T_H_17/IL17A‐mediated immunity.^[^ ^27^, ^28^, ^63^, ^64^, ^65^, ^66^ ^]^ We and others have shown that SFB contributes to commensal microbiota actions on skeletal growth and maturation at noncraniofacial sites. SFB induction of T_H_17/IL17A‐mediated immunity promoted bone‐resorbing osteoclasts and impaired trabecular bone morphology in the long bones of growing mice.^[^ ^13^, ^14^ ^]^ Further, prior reports linked SFB induction of T_H_17/IL17A immunity to commensal microbiota actions in neurodevelopmental abnormalities.^[^ ^29^, ^30^ ^]^ Therefore, commensal gut microbe contributions to craniofacial skeletal growth and morphology could be mediated through multifactorial effects on bone cell actions and brain development.

While SFB has been shown to colonize humans and upregulate T_H_17 cell immune pathway genes,^[^ ^67^, ^68^, ^69^ ^]^ *Bifidobacterium adolescentis* is another commensal gut bacterium that has been linked to the clinical induction of T_H_17/IL17A‐mediated immunity.^[^ ^70^ ^]^ Dietary modification^[^ ^71^, ^72^ ^]^ and probiotic administration^[^ ^73^, ^74^ ^]^ impact SFB colonization status, which implies that noninvasive interventions in the commensal gut microbiota could be employed to modify craniofacial skeletal growth and morphology. While randomized controlled clinical trials have shown that probiotics have protective effects on the aging skeleton and brain,^[^ ^75^, ^76^ ^]^ future investigations are needed to show whether probiotics could be employed to direct craniofacial skeletal growth and morphology.

Our study comparing EF versus MPF mice, which differ by a known commensal gut bacterium, supports the premise that commensal gut microbes play a role in craniofacial development. However, future research is necessary to further determine how specific microbiota communities (i.e., oral, gut) contribute to craniofacial skeletal morphology. Conventionalized animal studies, in which GF animals are associated/colonized with oral microbiota versus gut microbiota from specific‐pathogen‐free mice, would delineate how different microbiota communities contribute to craniofacial skeletal growth and maturation.

## Author Contributions

**Joy E. Gerasco:** Conceptualization; data curation; formal analysis; funding acquisition; investigation; methodology; software; writing – original draft; writing – review and editing. **Jessica D. Hathaway‐Schrader:** Data curation; formal analysis; investigation; writing – review and editing. **Nicole A. Poulides:** Data curation; investigation; writing – review and editing. **Matthew D. Carson:** Data curation; formal analysis; investigation; writing – review and editing. **Naoto Okhura:** Methodology; writing – review and editing. **Caroline Westwater:** Conceptualization; investigation; methodology; writing – review and editing. **Nan E Hatch:** Conceptualization; methodology; supervision; writing – review and editing. **Chad M. Novince:** Conceptualization; formal analysis; funding acquisition; investigation; methodology; resources; software; supervision; writing – original draft; writing – review and editing.

## Funding Information

This work was funded by an American Society for Bone and Mineral Research Rising Star Award, K08DE025337, T32DE017551, P20GM130457, P30DK123704, P20GM121342, R01DE029637, R01AG067510, UL1TR001450, F30DE027290, R01AR081488.

## Disclosures

The authors declare no conflicts of interest.

### Peer Review

The peer review history for this article is available at https://www.webofscience.com/api/gateway/wos/peer-review/10.1002/jbm4.10775.

## Supporting information


**Fig. S1.** Craniometric landmarks and linear measurements.
**Fig. S2.** Commensal microbiota does not affect cranial bone mineral density in 9‐week‐old female C57BL/6T mice. Female C57BL/6T GF mice (no microbes), EF mice (commensal microbiota), and MPF mice (commensal microbiota with SFB) were euthanized at age 9‐weeks; skulls were isolated for analyses (*n* = 5/group). Bone mineral density (BMD) analyses were performed in μCT 3D reconstructions of the skull, using defined volumes of interest (red boxes) for cranial vault bones (***A‐D***) and cranial base bones (***E‐H***). BMD outcomes for interparietal bone (***B***), parietal bone (***C***), frontal bone (***D***), basioccipital bone (***F***), basisphenoid bone (***G***), and presphenoid bone (***H***). One‐way ANOVA (α = 0.05) with Tukey post hoc test (*P* < 0.05) was carried out comparing outcomes in GF, EF, and MPF mice.

## Data Availability

The data used and/or analyzed during the current study are available from the corresponding author upon request.

## References

[1] Belkaid Y , Naik S . Compartmentalized and systemic control of tissue immunity by commensals. Nat Immunol. 2013;14(7):646–653. 23778791 10.1038/ni.2604 PMC3845005

[2] Brestoff JR , Artis D . Commensal bacteria at the interface of host metabolism and the immune system. Nat Immunol. 2013;14(7):676–684. 23778795 10.1038/ni.2640 PMC4013146

[3] Thaiss CA , Zmora N , Levy M , Elinav E . The microbiome and innate immunity. Nature. 2016;535(7610):65–74. 27383981 10.1038/nature18847

[4] Zheng D , Liwinski T , Elinav E . Interaction between microbiota and immunity in health and disease. Cell Res. 2020;30(6):492–506. 32433595 10.1038/s41422-020-0332-7 PMC7264227

[5] Sommer F , Backhed F . The gut microbiota–masters of host development and physiology. Nat Rev Microbiol. 2013;11(4):227–238. 23435359 10.1038/nrmicro2974

[6] Schroeder BO , Bäckhed F . Signals from the gut microbiota to distant organs in physiology and disease. Nat Med. 2016;22(10):1079–1089. 27711063 10.1038/nm.4185

[7] Krautkramer KA , Fan J , Bäckhed F . Gut microbial metabolites as multi‐kingdom intermediates. Nat Rev Microbiol. 2021;19(2):77–94. 32968241 10.1038/s41579-020-0438-4

[8] Fan Y , Pedersen O . Gut microbiota in human metabolic health and disease. Nat Rev Microbiol. 2021;19(1):55–71. 32887946 10.1038/s41579-020-0433-9

[9] Sjögren K , Engdahl C , Henning P , et al. The gut microbiota regulates bone mass in mice. J Bone Miner Res. 2012;27(6):1357–1367. 22407806 10.1002/jbmr.1588 PMC3415623

[10] Novince CM , Whittow CR , Aartun JD , et al. Commensal gut microbiota immunomodulatory actions in bone marrow and liver have catabolic effects on skeletal homeostasis in health. Sci Rep. 2017;7(1):5747. 28720797 10.1038/s41598-017-06126-x PMC5515851

[11] Schwarzer M , Makki K , Storelli G , et al. *Lactobacillus plantarum* strain maintains growth of infant mice during chronic undernutrition. Science. 2016;351(6275):854–857. 26912894 10.1126/science.aad8588

[12] Yan J , Herzog JW , Tsang K , et al. Gut microbiota induce IGF‐1 and promote bone formation and growth. Proc Natl Acad Sci U.S.A. 2016;113(47):E7554–E7563. 27821775 10.1073/pnas.1607235113 PMC5127374

[13] Hathaway‐Schrader JD , Poulides NA , Carson MD , et al. Specific commensal bacterium critically regulates gut microbiota osteoimmunomodulatory actions during normal postpubertal skeletal growth and maturation. JBMR Plus. 2020;4(3):e10338. 32161843 10.1002/jbm4.10338 PMC7059828

[14] Tyagi AM , Darby TM , Hsu E , et al. The gut microbiota is a transmissible determinant of skeletal maturation. Elife. 2021;10:e64237. 33432923 10.7554/eLife.64237 PMC7803376

[15] Galea GL , Zein MR , Allen S , Francis‐West P . Making and shaping endochondral and intramembranous bones. Dev Dyn. 2021;250(3):414–449. 33314394 10.1002/dvdy.278 PMC7986209

[16] White HE , Goswami A , Tucker AS . The intertwined evolution and development of sutures and cranial morphology. Front Cell Dev Biol. 2021;9:653579. 33842480 10.3389/fcell.2021.653579 PMC8033035

[17] Lacruz RS , Stringer CB , Kimbel WH , et al. The evolutionary history of the human face. Nat Ecol Evol. 2019;3(5):726–736. 30988489 10.1038/s41559-019-0865-7

[18] Park KM , Tripathi NV , Mufarrej FA . Quality of life in patients with craniosynostosis and deformational plagiocephaly: a systematic review. Int J Pediatr Otorhinolaryngol. 2021;149:110873. 34380097 10.1016/j.ijporl.2021.110873

[19] Kawakami M , Yamamura K . Cranial bone morphometric study among mouse strains. BMC Evol Biol. 2008;8:73. 18307817 10.1186/1471-2148-8-73 PMC2287174

[20] Vora SR , Camci ED , Cox TC . Postnatal ontogeny of the cranial base and craniofacial skeleton in male C57BL/6J mice: a reference standard for quantitative analysis. Front Physiol. 2015;6:417. 26793119 10.3389/fphys.2015.00417 PMC4709510

[21] Wei X , Thomas N , Hatch NE , Hu M , Liu F . Postnatal craniofacial skeletal development of female C57BL/6NCrl mice. Front Physiol. 2017;8:697. 28959213 10.3389/fphys.2017.00697 PMC5603710

[22] Young DL , Schneider RA , Hu D , Helms JA . Genetic and teratogenic approaches to craniofacial development. Crit Rev Oral Biol Med. 2000;11(3):304–317. 11021632 10.1177/10454411000110030201

[23] Neben CL , Merrill AE . Signaling pathways in craniofacial development: insights from rare skeletal disorders. Curr Top Dev Biol. 2015;115:493–542. 26589936 10.1016/bs.ctdb.2015.09.005

[24] Stanton E , Urata M , Chen JF , Chai Y . The clinical manifestations, molecular mechanisms and treatment of craniosynostosis. Dis Model Mech. 2022;15(4):1–18. 10.1242/dmm.049390 PMC9044212 35451466

[25] Klaasen HL , Koopman JP , Beynen AC . Effects of age, strain and social hierarchy on colonization of autochthonous, segmented, filamentous bacteria in the ileum of mice. Microecol Therapy. 1990b;20:17–20.

[26] Snel J , Hermsen CC , Smits HJ , et al. Interactions between gut‐associated lymphoid tissue and colonization levels of indigenous, segmented, filamentous bacteria in the small intestine of mice. Can J Microbiol. 1998;44(12):1177–1182. 10347864 10.1139/cjm-44-12-1177

[27] Gaboriau‐Routhiau V , Rakotobe S , Lecuyer E , et al. The key role of segmented filamentous bacteria in the coordinated maturation of gut helper T cell responses. Immunity. 2009;31(4):677–689. 19833089 10.1016/j.immuni.2009.08.020

[28] Ivanov II , Atarashi K , Manel N , et al. Induction of intestinal Th17 cells by segmented filamentous bacteria. Cell. 2009;139(3):485–498. 19836068 10.1016/j.cell.2009.09.033 PMC2796826

[29] Kim S , Kim H , Yim YS , et al. Maternal gut bacteria promote neurodevelopmental abnormalities in mouse offspring. Nature. 2017;549(7673):528–532. 28902840 10.1038/nature23910 PMC5870873

[30] Lammert CR , Frost EL , Bolte AC , et al. Cutting edge: critical roles for microbiota‐mediated regulation of the immune system in a prenatal immune activation model of autism. J Immunol. 2018;201(3):845–850. 29967099 10.4049/jimmunol.1701755 PMC6057827

[31] Massimi L , Bianchi F , Frassanito P , Calandrelli R , Tamburrini G , Caldarelli M . Imaging in craniosynostosis: when and what? Childs Nerv Syst. 2019;35(11):2055–2069. 31289853 10.1007/s00381-019-04278-x

[32] Dot G , Rafflenbeul F , Arbotto M , Gajny L , Rouch P , Schouman T . Accuracy and reliability of automatic three‐dimensional cephalometric landmarking. Int J Oral Maxillofac Surg. 2020;49(10):1367–1378. 32169306 10.1016/j.ijom.2020.02.015

[33] Council NR . Guide for the care and use of laboratory animals. Eighth ed. Washington, DC: The National Academies Press; 2011. 21595115

[34] Bacchetti De Gregoris T , Aldred N , Clare AS , Burgess JG . Improvement of phylum‐ and class‐specific primers for real‐time PCR quantification of bacterial taxa. J Microbiol Methods. 2011;86(3):351–356. 21704084 10.1016/j.mimet.2011.06.010

[35] Hathaway‐Schrader JD , Steinkamp HM , Chavez MB , et al. Antibiotic perturbation of gut microbiota dysregulates osteoimmune cross talk in postpubertal skeletal development. Am J Pathol. 2019;189(2):370–390. 30660331 10.1016/j.ajpath.2018.10.017 PMC6360355

[36] Swanson BA , Carson MD , Hathaway‐Schrader JD , et al. Antimicrobial‐induced oral dysbiosis exacerbates naturally occurring alveolar bone loss. FASEB J. 2021;35(11):e22015. 34699641 10.1096/fj.202101169R PMC8732259

[37] Packey CD , Shanahan MT , Manick S , et al. Molecular detection of bacterial contamination in gnotobiotic rodent units. Gut Microbes. 2013;4(5):361–370. 23887190 10.4161/gmic.25824 PMC3839980

[38] Hathaway‐Schrader JD , Aartun JD , Poulides NA , et al. Commensal oral microbiota induces osteoimmunomodulatory effects separate from systemic microbiome in mice. JCI Insight. 2022;7(4):1–20. 10.1172/jci.insight.140738 PMC8876522 35077397

[39] Livak KJ , Schmittgen TD . Analysis of relative gene expression data using real‐time quantitative PCR and the 2(‐Delta Delta C(T)) method. Methods. 2001;25(4):402–408. 11846609 10.1006/meth.2001.1262

[40] Schmittgen TD , Livak KJ . Analyzing real‐time PCR data by the comparative C(T) method. Nat Protoc. 2008;3(6):1101–1108. 18546601 10.1038/nprot.2008.73

[41] Hathaway‐Schrader JD , Carson MD , Gerasco JE , et al. Commensal gut bacterium critically regulates alveolar bone homeostasis. Lab Invest. 2022;102(4):363–375. 34934182 10.1038/s41374-021-00697-0 PMC8967765

[42] Liu J , Nam HK , Campbell C , Gasque KC , Millán JL , Hatch NE . Tissue‐nonspecific alkaline phosphatase deficiency causes abnormal craniofacial bone development in the Alpl(−/−) mouse model of infantile hypophosphatasia. Bone. 2014;67:81–94. 25014884 10.1016/j.bone.2014.06.040 PMC4149826

[43] Xu H , Lenhart SA , Chu EY , et al. Dental and craniofacial defects in the Crtap(−/−) mouse model of osteogenesis imperfecta type VII. Dev Dyn. 2020;249(7):884–897. 32133710 10.1002/dvdy.166 PMC7727892

[44] Nam HK , Emmanouil E , Hatch NE . Deletion of the pyrophosphate generating enzyme ENPP1 rescues craniofacial abnormalities in the TNAP(−/−) mouse model of Hypophosphatasia and reveals FGF23 as a marker of phenotype severity. Front Dent Med. 2022;3:1–14. 10.3389/fdmed.2022.846962 PMC9336114 35909501

[45] Bouxsein ML , Boyd SK , Christiansen BA , Guldberg RE , Jepsen KJ , Müller R . Guidelines for assessment of bone microstructure in rodents using micro‐computed tomography. J Bone Miner Res. 2010;25(7):1468–1486. 20533309 10.1002/jbmr.141

[46] Liu J , Nam HK , Wang E , Hatch NE . Further analysis of the Crouzon mouse: effects of the FGFR2(C342Y) mutation are cranial bone‐dependent. Calcif Tissue Int. 2013;92(5):451–466. 23358860 10.1007/s00223-013-9701-2 PMC3631296

[47] Liu J , Campbell C , Nam HK , et al. Enzyme replacement for craniofacial skeletal defects and craniosynostosis in murine hypophosphatasia. Bone. 2015;78:203–211. 25959417 10.1016/j.bone.2015.05.005 PMC4466206

[48] Funato N . New insights into cranial synchondrosis development: a mini review. Front Cell Dev Biol. 2020;8:706. 32850826 10.3389/fcell.2020.00706 PMC7432265

[49] Bastir M . Craniofacial levels and the morphological maturation of the human skull. J Anat. 2006;209:637–654. 17062021 10.1111/j.1469-7580.2006.00644.x PMC2100348

[50] Gong A , Li J , Wang Z , et al. Cranial base characteristics in anteroposterior malocclusions: a meta‐analysis. Angle Orthod. 2016;86(4):668–680. 26528732 10.2319/032315-186.1 PMC8601493

[51] Almeida KCM , Raveli TB , Vieira CIV , Santos‐Pinto AD , Raveli DB . Influence of the cranial base flexion on class I, II and III malocclusions: a systematic review. Dental Press J Orthod. 2017;22(5):56–66. 10.1590/2177-6709.22.5.056-066.oar PMC5730137 29160345

[52] Kiliaridis S . The importance of masticatory muscle function in dentofacial growth. Semin Orthod. 2006;12(2):110–119.

[53] Kono K , Tanikawa C , Murata Y , Yanagita T , Kamioka H , Yamashiro T . Three‐dimensional changes in the craniofacial complex associated with soft‐diet feeding. Eur J Orthod. 2020;42(5):509–516. 32346737 10.1093/ejo/cjaa007

[54] Heijtz RD , Wang S , Anuar F , et al. Normal gut microbiota modulates brain development and behavior. Proc Natl Acad Sci U.S.A. 2011;108(7):3047–3052. 21282636 10.1073/pnas.1010529108 PMC3041077

[55] Hoban AE , Stilling RM , Ryan FJ , et al. Regulation of prefrontal cortex myelination by the microbiota. Transl Psychiatry. 2016;6(4):e774. 27045844 10.1038/tp.2016.42 PMC4872400

[56] Lu J , Synowiec S , Lu L , et al. Microbiota influence the development of the brain and behaviors in C57BL/6J mice. PLoS One. 2018;13(8):e0201829. 30075011 10.1371/journal.pone.0201829 PMC6075787

[57] Ahmed S , Travis SD , Díaz‐Bahamonde FV , et al. Early influences of microbiota on white matter development in germ‐free piglets. Front Cell Neurosci. 2021;15:807170. 35027884 10.3389/fncel.2021.807170 PMC8751630

[58] Moss ML , Young RW . A functional approach to craniology. Am J Phys Anthropol. 1960;18(4):281–292. 13773136 10.1002/ajpa.1330180406

[59] Lieberman DE , Pearson OM , Mowbray KM . Basicranial influence on overall cranial shape. J Hum Evol. 2000;38(2):291–315. 10656780 10.1006/jhev.1999.0335

[60] Hallgrímsson B , Lieberman DE . Mouse models and the evolutionary developmental biology of the skull. Integr Comp Biol. 2008;48(3):373–384. 21669799 10.1093/icb/icn076

[61] Marcucio RS , Young NM , Hu D , Hallgrimsson B . Mechanisms that underlie co‐variation of the brain and face. Genesis. 2011;49(4):177–189. 21381182 10.1002/dvg.20710 PMC3086711

[62] Adameyko I , Fried K . The nervous system orchestrates and integrates craniofacial development: a review. Front Physiol. 2016;7:49. 26924989 10.3389/fphys.2016.00049 PMC4759458

[63] Goto Y , Panea C , Nakato G , et al. Segmented filamentous bacteria antigens presented by intestinal dendritic cells drive mucosal Th17 cell differentiation. Immunity. 2014;40(4):594–607. 24684957 10.1016/j.immuni.2014.03.005 PMC4084624

[64] Lecuyer E , Rakotobe S , Lengline‐Garnier H , et al. Segmented filamentous bacterium uses secondary and tertiary lymphoid tissues to induce gut IgA and specific T helper 17 cell responses. Immunity. 2014;40(4):608–620. 24745335 10.1016/j.immuni.2014.03.009

[65] Panea C , Farkas AM , Goto Y , et al. Intestinal monocyte‐derived macrophages control commensal‐specific Th17 responses. Cell Rep. 2015;12(8):1314–1324. 26279572 10.1016/j.celrep.2015.07.040 PMC4567384

[66] Flannigan KL , Ngo VL , Geem D , et al. IL‐17A‐mediated neutrophil recruitment limits expansion of segmented filamentous bacteria. Mucosal Immunol. 2017;10(3):673–684. 27624780 10.1038/mi.2016.80 PMC5350071

[67] Yin Y , Wang Y , Zhu L , et al. Comparative analysis of the distribution of segmented filamentous bacteria in humans, mice and chickens. ISME J. 2013;7(3):615–621. 23151642 10.1038/ismej.2012.128 PMC3578561

[68] Chen B , Chen H , Shu X , et al. Presence of segmented filamentous bacteria in human children and its potential role in the modulation of human gut immunity. Front Microbiol. 2018;9:1403. 30008704 10.3389/fmicb.2018.01403 PMC6034559

[69] Chen H , Wang L , Wang X , Wang X , Liu H , Yin Y . Distribution and strain diversity of Immunoregulating segmented filamentous bacteria in human intestinal lavage samples. Microb Ecol. 2020;79(4):1021–1033. 31728601 10.1007/s00248-019-01441-4

[70] Tan TG , Sefik E , Geva‐Zatorsky N , et al. Identifying species of symbiont bacteria from the human gut that, alone, can induce intestinal Th17 cells in mice. Proc Natl Acad Sci U.S.A. 2016;113(50):E8141–E8150. 27911839 10.1073/pnas.1617460113 PMC5167147

[71] Koopman JP , van den Brink ME , Scholten PM . Further studies with the segmented filamentous intestinal bacteria of mice; effects of physical and chemical factors on survival and the effects of milk diet, para‐aminobenzoic acid and mouse strain on colonization. Zeitschrift fur Versuchstierkunde. 1988;31(6):270–275. 3265829

[72] Koopman JP , van den Brink ME , Scholtenl PM , et al. The influence of stress and cheese‐whey on intestinal parameters in mice. Vet Q. 1989;11(1):24–29. 2655262 10.1080/01652176.1989.9694192

[73] Fuentes S , Egert M , Jimenez‐Valera M , Monteoliva‐Sanchez M , Ruiz‐Bravo A , Smidt H . A strain of *Lactobacillus plantarum* affects segmented filamentous bacteria in the intestine of immunosuppressed mice. FEMS Microbiol Ecol. 2008;63(1):65–72. 18081591 10.1111/j.1574-6941.2007.00411.x

[74] Liao N , Yin Y , Sun G , et al. Colonization and distribution of segmented filamentous bacteria (SFB) in chicken gastrointestinal tract and their relationship with host immunity. FEMS Microbiol Ecol. 2012;81(2):395–406. 22429007 10.1111/j.1574-6941.2012.01362.x

[75] Nilsson AG , Sundh D , Bäckhed F , Lorentzon M . *Lactobacillus reuteri* reduces bone loss in older women with low bone mineral density: a randomized, placebo‐controlled, double‐blind, clinical trial. J Intern Med. 2018;284(3):307–317. 29926979 10.1111/joim.12805

[76] Kim CS , Cha L , Sim M , et al. Probiotic supplementation improves cognitive function and mood with changes in gut microbiota in community‐dwelling older adults: a randomized, double‐blind, placebo‐controlled, multicenter trial. J Gerontol A Biol Sci Med Sci. 2021;76(1):32–40. 32300799 10.1093/gerona/glaa090 PMC7861012

