# Supplementary material for: Commensal Microbiota Effects on Craniofacial Skeletal Growth and Morphology
Source: JBMR Plus. 2023 May 31;7(8):e10775. doi: 10.1002/jbm4.10775 (PMC10443078; doi:10.1002/jbm4.10775)
Supplement: Supplementary file 1 — Fig. S1. Craniometric landmarks and linear measurements. Fig. S2. Commensal microbiota does not affect cranial bone mineral density in 9‐week‐old female C57BL/6T mice. Female C57BL/6T GF mice (no microbes), EF mice (commensal microbiota), and MPF mice (commensal microbiota with SFB) were euthanized at age 9‐weeks; skulls were isolated for analyses (n = 5/group). Bone mineral density (BMD) analyses were performed in μCT 3D reconstructions of the skull, using defined volumes of interest (red boxes) for cranial vault bones ( A‐D ) and cranial base bones ( E‐H ). BMD outcomes for interparietal bone ( B ), parietal bone ( C ), frontal bone ( D ), basioccipital bone ( F ), basisphenoid bone ( G ), and presphenoid bone ( H ). One‐way ANOVA (α = 0.05) with Tukey post hoc test (P < 0.05) was carried out comparing outcomes in GF, EF, and MPF mice. [file JBM4-7-e10775-s001.docx]

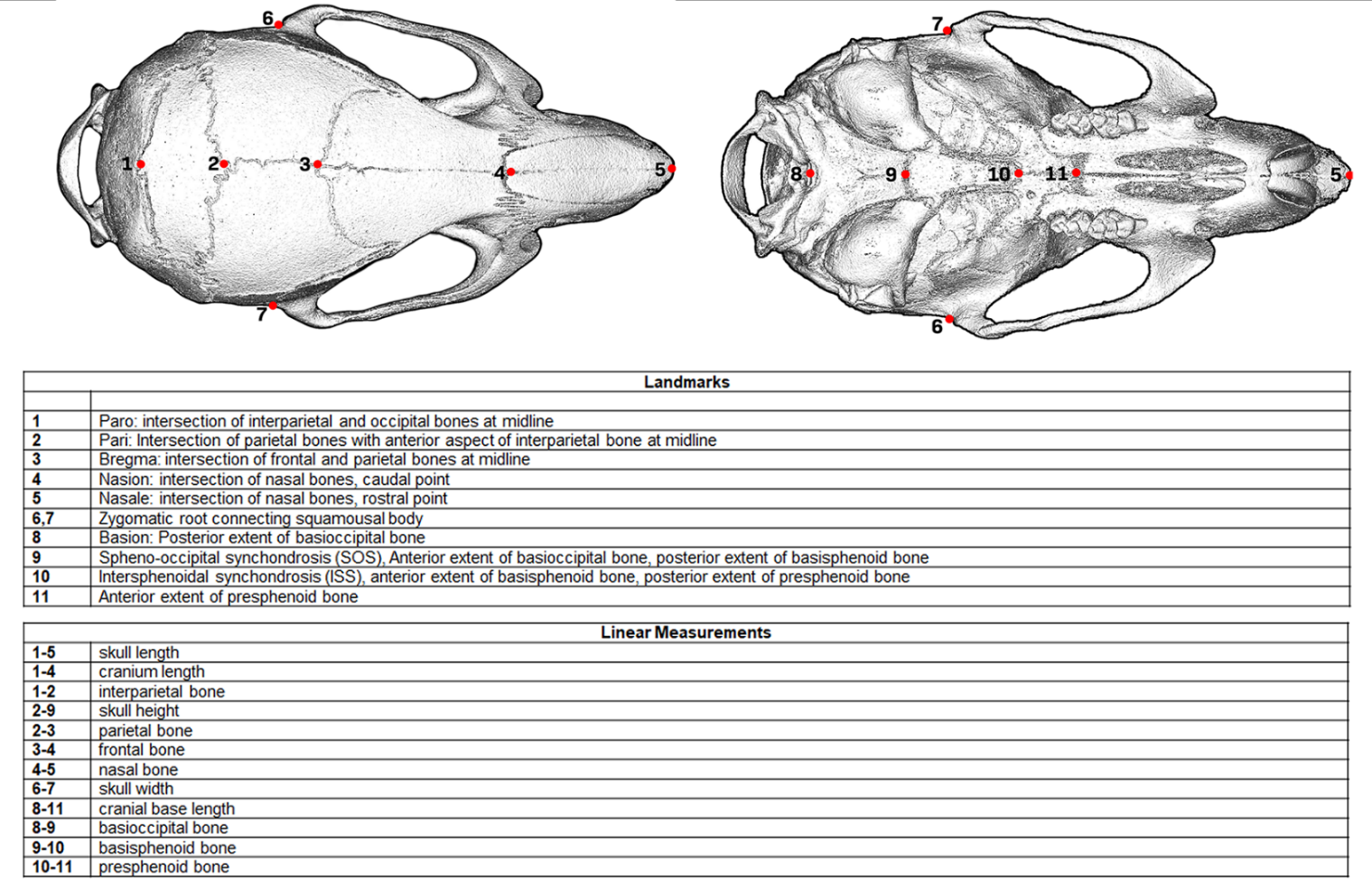


| Linear Measurements | |
| --- | --- |
| **1-5** | skull length |
| **1-4** | cranial vault length |
| **1-2** | interparietal bone length |
| **2-9** | skull height |
| **2-3** | parietal bone length |
| **3-4** | frontal bone length |
| **4-5** | nasal bone length |
| **6-7** | skull width |
| **8-11** | cranial base length |
| **8-9** | basioccipital bone length |
| **9-10** | basisphenoid bone length |
| **10-11** | presphenoid bone length |

| Landmarks | |
| --- | --- |
| **1** | Paro: intersection of interparietal and occipital bones at midline |
| **2** | Pari: intersection of parietal bones with anterior aspect of interparietal bone at midline |
| **3** | Bregma: intersection of frontal and parietal bones at midline |
| **4** | Nasion: intersection of nasal bones, caudal point |
| **5** | Nasale: intersection of nasal bones, rostral point |
| **6,7** | Zygomatic root connecting squamosal body |
| **8** | Basion: posterior extent of basioccipital bone |
| **9** | Spheno-occipital synchondrosis (SOS), Anterior extent of basioccipital bone, posterior extent of basisphenoid bone |
| **10** | Intersphenoidal synchondrosis (ISS), anterior extent of basisphenoid bone, posterior extent of presphenoid bone |
| **11** | Anterior extent of presphenoid bone |

**Supplemental Figure S1. Craniometric landmarks and linear measurements.**


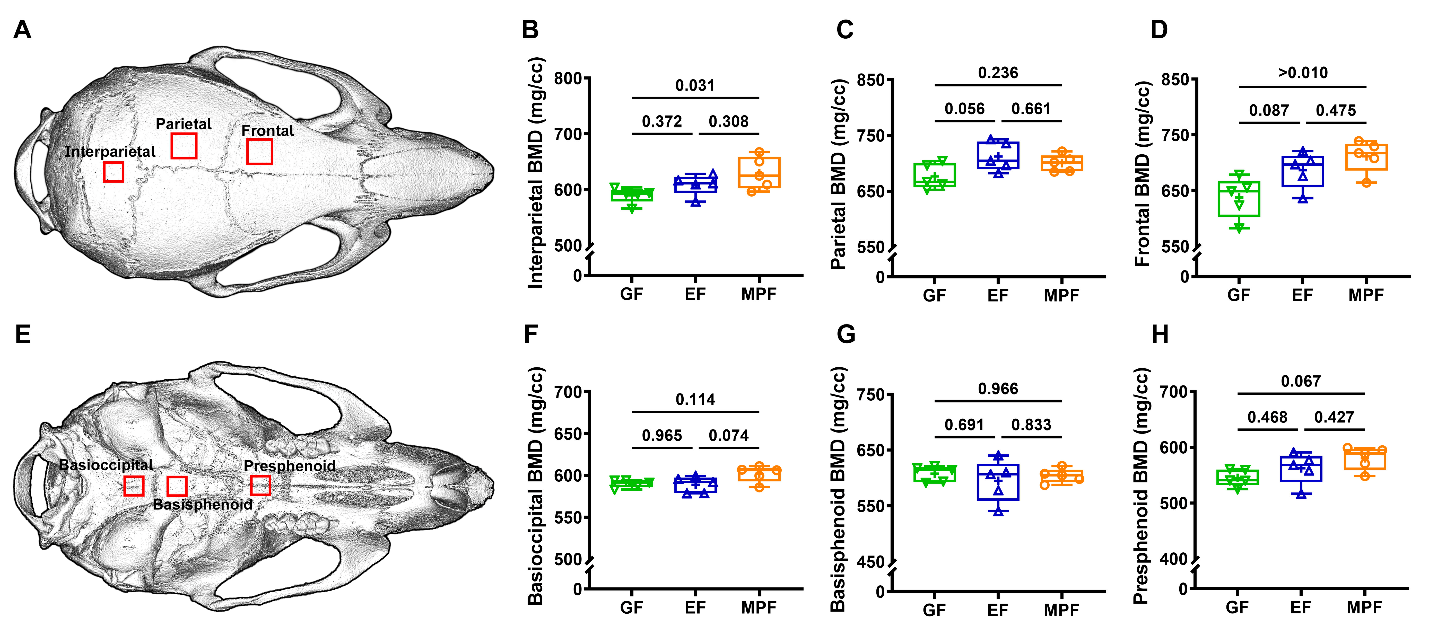
**Supplemental Figure S2. Commensal microbiota does not affect cranial bone mineral density in 9-week-old female C57BL/6T mice.** Female C57BL/6T GF mice (no microbes), EF mice (commensal microbiota), and MPF mice (commensal microbiota with SFB) were euthanized at age 9-weeks; skulls were isolated for analyses (n=5/group). Bone mineral density (BMD) analyses were performed in μCT 3D reconstructions of the skull, using defined volumes of interest (red boxes) for cranial vault bones (***A-D***) and cranial base bones (***E-H***). BMD outcomes for interparietal bone (***B***), parietal bone (***C***), frontal bone (***D***), basioccipital bone (***F***), basisphenoid bone (***G***), and presphenoid bone (***H***). One-way ANOVA (α = 0.05) with Tukey post hoc test (P < 0.05) was carried out comparing outcomes in GF, EF, and MPF mice.
